# 18F-Fluorodeoxyglucose (FDG) PET/CT Follow-Up in Radioiodine-Refractory Papillary Thyroid Carcinoma: A Case Report

**DOI:** 10.7759/cureus.102593

**Published:** 2026-01-29

**Authors:** David Gutierrez Albenda, Valeria Armani Arce, Ernesto Balmaceda Araya, Akira Osawa Pivovarov, Álvaro Montoya Porras

**Affiliations:** 1 Cyclotron-PET/CT Laboratory, University of Costa Rica, San José, CRI; 2 School of Medicine, University of Costa Rica, San José, CRI

**Keywords:** 18f-fdg pet/ct, follow up, iodine therapy, thyroid papillary carcinoma, thyroid radioiodine-refractory cancer

## Abstract

Thyroid carcinoma is the most frequent endocrine malignancy, and papillary thyroid carcinoma (PTC) is its predominant subtype. Standard initial management includes surgical resection followed by radioactive iodine (¹³¹I) ablation. PET with fluorodeoxyglucose (¹⁸F-FDG) combined with CT (FDG-PET/CT) has emerged as a valuable tool during follow-up, particularly in cases of radioiodine-refractory disease. We report the case of a 67-year-old man with PTC refractory to multiple ¹³¹I therapies, whose disease progression was monitored using serial FDG-PET/CT imaging. This case highlights the role of FDG-PET/CT in guiding treatment selection in advanced PTC.

## Introduction

Thyroid cancer is the most common endocrine malignancy, and its incidence has been rising steadily. It is classified into three main categories: differentiated cancer (which includes papillary, follicular, and oncocytic carcinoma), medullary carcinoma, and anaplastic carcinoma [1]. Among these, papillary thyroid carcinoma is the most frequent subtype. It arises from follicular cells and is characterized histologically by the formation of papillary structures [2].

The standard treatment consists of surgical resection followed by ablation therapy with radioactive iodine (¹³¹I). Patient follow-up is typically performed using serum thyroglobulin measurements and cervical ultrasound. However, additional imaging modalities, such as 18F-fluorodeoxyglucose (FDG-PET/CT) and MRI, are also employed in selected cases [3].

FDG-PET/CT is based on the use of the radiotracer fluorodeoxyglucose (¹⁸F-FDG), a glucose analog [4]. This tracer enters cells through GLUT transporters but cannot be metabolized beyond FDG-6-phosphate. As a result, it accumulates intracellularly, reflecting cellular metabolic activity. Poorly differentiated tumor cells often overexpress glycolytic enzymes and glucose transporters, making FDG-PET/CT particularly useful for cancer detection, staging, and assessment of recurrence [5].

We report the case of a 67-year-old man diagnosed with papillary thyroid carcinoma who initially underwent thyroidectomy followed by multiple courses of ¹³¹I. Despite therapy, he continued to have elevated thyroglobulin levels and was therefore classified as having radioiodine-refractory disease. Radioiodine-refractory disease is defined as the inability of differentiated thyroid cancer to concentrate ¹³¹I at the time of initial treatment or after initial therapy, which leads to disease progression and a poor prognosis [6]. He subsequently received treatment with tyrosine kinase inhibitors (TKIs). During his clinical course, FDG-PET/CT was employed and revealed nodal and bone metastatic progression. This case report highlights the usefulness of FDG-PET/CT as an imaging method in the follow-up and evaluation of radioiodine-refractory differentiated thyroid cancer, enabling a more precise assessment of disease status and progression and contributing to the selection of targeted therapy.

## Case presentation

A 67-year-old man with a history of hypertension, keratoconus, and benign prostatic hyperplasia was diagnosed in May 2020 with papillary thyroid carcinoma following fine-needle aspiration (FNA) of a right thyroid nodule and lymph nodes at levels III, IV, and VI. The patient underwent total thyroidectomy with lymphadenectomy in June 2020. Staging studies revealed pulmonary metastases, and he subsequently received three courses of ¹³¹I therapy: October 3, 2020 (148 mCi), June 21, 2021 (152 mCi), and April 8, 2022 (202 mCi), for a total cumulative dose of 502 mCi. After ¹³¹I therapy, thyroglobulin levels were monitored and remained markedly elevated: 802.9 ng/mL (June 24, 2022), 1038.9 ng/mL (August 24, 2022), and 1038 ng/mL (October 5, 2022).

Despite these treatments, serum thyroglobulin levels remained persistently elevated. In this context, the persistent elevation of thyroglobulin despite multiple courses of ¹³¹I suggests an inability to concentrate ¹³¹I, consistent with radioiodine-refractory differentiated thyroid cancer. Therapy with sorafenib 800 mg daily was initiated on December 5, 2022; subsequently, the patient developed toxicities including plantar erythrodysesthesia, xerosis, alopecia, stomatitis, arterial hypertension, and hemorrhoids, which were managed with esomeprazole, telmisartan, and topical treatments.

A CT scan performed on April 26, 2023, showed two right level V cervical lymph nodes and innumerable pulmonary nodules measuring 2-3 mm, consistent with persistent metastatic disease.

**Figure 1:**
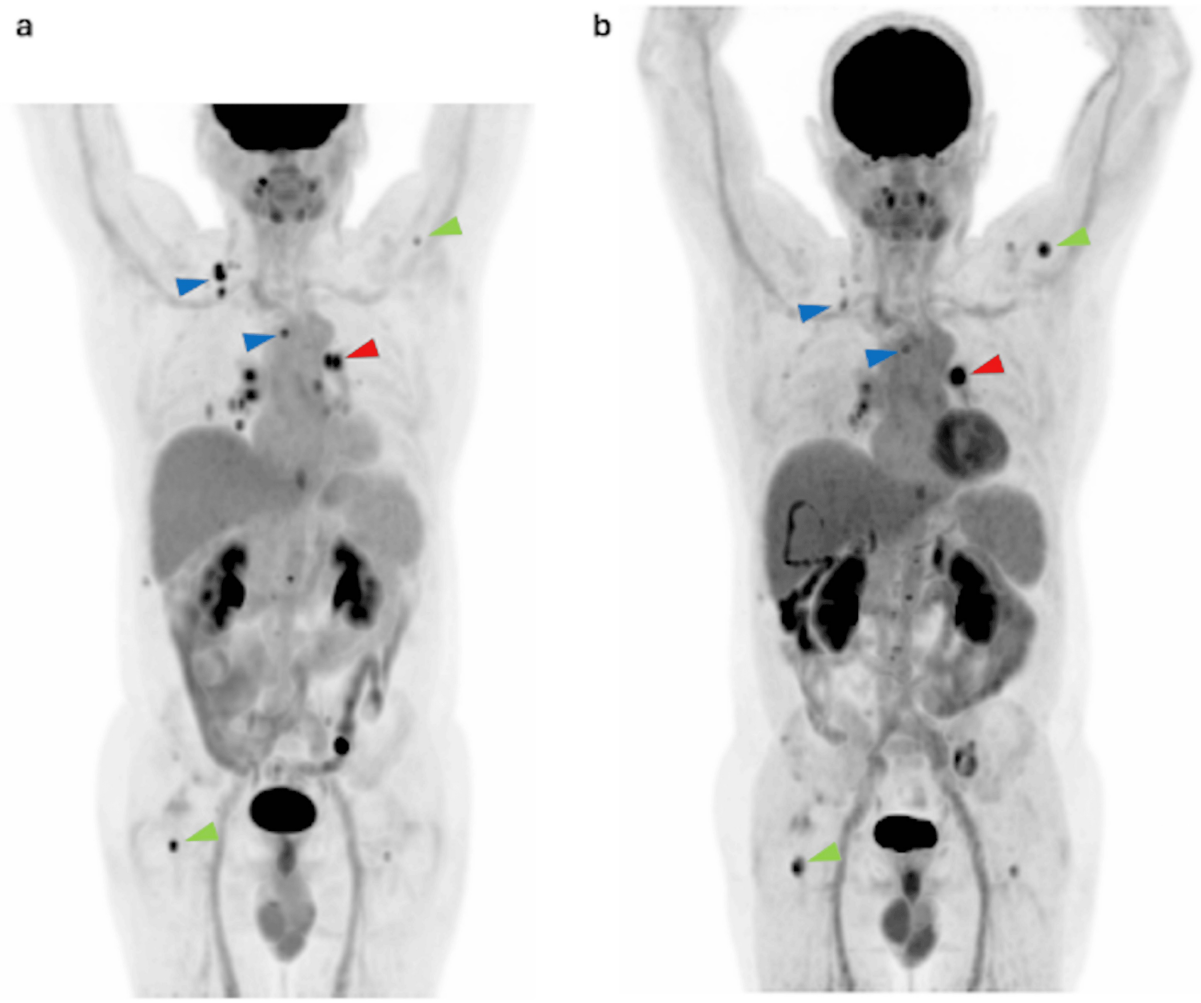
MIP images from FDG-PET/CT studies performed in June 2023 (a) and July 2024 (b). Interval metabolic progression is observed, characterized by increased FDG uptake in a left hilar pulmonary lymph node (red arrowhead) and multiple osseous lesions (green arrowhead), while several cervical and mediastinal lymph nodes show decreased tracer avidity, indicating a mixed metabolic response (blue arrowhead). MIP: Maximum intensity projection; FDG: Fluorodeoxyglucose.

FDG-PET/CT performed on June 13, 2023, demonstrated hypermetabolic disease involving the right posterior cervical region (lymphadenopathy), bilateral lungs, and multiple bone sites (Figure 1).

Sorafenib was discontinued, and the patient was placed under surveillance. A repeat FDG-PET/CT on September 7, 2023, revealed mild nodal and osseous progression. Thyroglobulin levels were 2284.77 ng/mL (May 15, 2023), 2517 ng/mL (June 20, 2023), and 4948 ng/mL (September 8, 2023). Based on these findings, combination therapy with dabrafenib and trametinib was initiated in October 2023.

**Figure 2:**
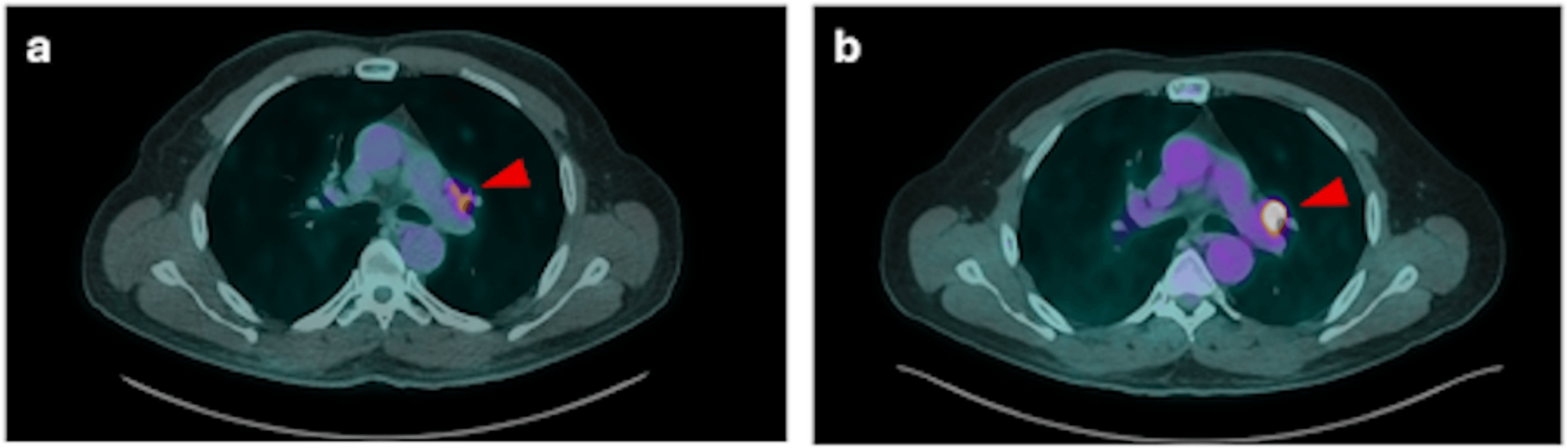
Axial FDG-PET/CT images from June 2023 (a) and July 2024 (b). An interval increase in the metabolic activity of a left hilar pulmonary nodal lesion (red arrowhead) indicates progressive disease. FDG: Fluorodeoxyglucose.

Follow-up thyroglobulin levels were 2732.07 ng/mL (November 2, 2023), 3402 ng/mL (January 5, 2024), and 3024 ng/mL (April 1, 2024). FDG-PET/CT on April 1, 2024, showed a partial metabolic response in cervical and mediastinal lymph nodes and pulmonary lesions, with a dissociated response in bone metastases (Figure 2).

**Figure 3:**
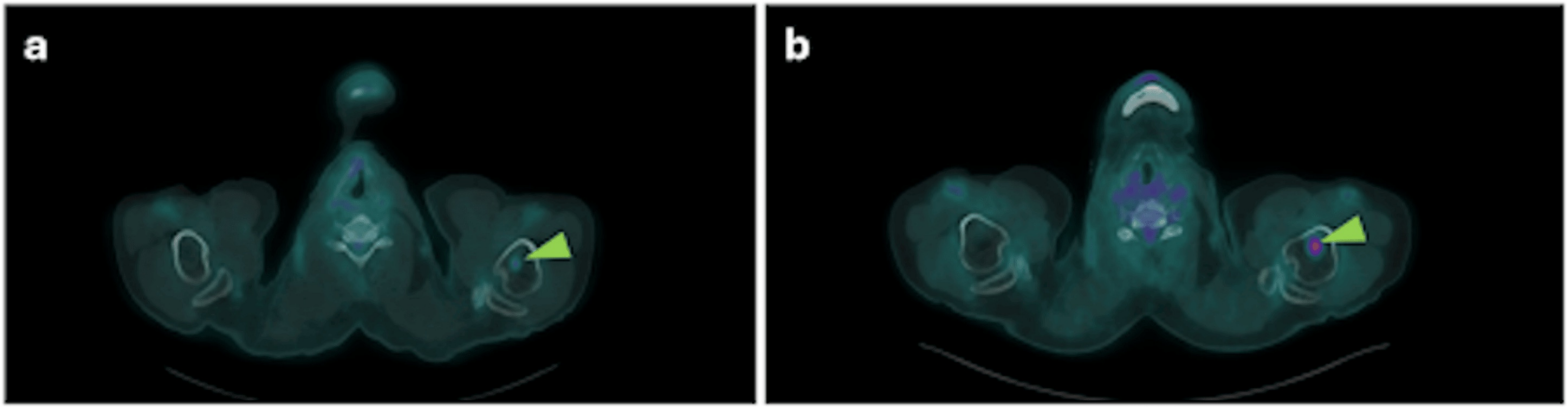
Axial FDG-PET/CT images from June 2023 (a) and July 2024 (b) demonstrate increased metabolic activity in a bone lesion in the proximal third of the left humerus (green arrowhead), compatible with disease progression. FDG: Fluorodeoxyglucose.

A subsequent PET/CT on July 9, 2024, demonstrated marked metabolic progression in a left hilar pulmonary lymph node, stable cervical and mediastinal disease, a mild increase in hypermetabolism of three pulmonary nodules, and slight progression of osseous lesions at T4, T12, and the left iliac bone (Figure 3). The patient was referred for continued multidisciplinary management.

## Discussion

We present the case of a 67-year-old man diagnosed with papillary thyroid carcinoma by FNA. Standard first-line management involves surgical resection, with the specific approach guided by risk stratification. In this case, the patient underwent total thyroidectomy with lymphadenectomy. Following surgery, ¹³¹I therapy was administered. Thyroid tissue has a high capacity for iodine uptake, and in the context of therapy, ¹³¹I is taken up and concentrated by follicular thyroid cells via the sodium-iodide symporter [7].

¹³¹I therapy is used as an adjuvant to total thyroidectomy in patients with well-differentiated thyroid cancer. It is administered orally as sodium iodide, typically 4-6 weeks after surgery. The therapeutic goal is to ablate residual thyroid tissue or to serve as targeted therapy for residual or metastatic disease [7,8]. Treatment efficacy is assessed by serum thyroglobulin levels, which should ideally remain low. In this patient, however, thyroglobulin levels remained persistently elevated even after multiple courses of ¹³¹I, consistent with radioiodine-refractory disease [8,9].

In this context, FDG-PET/CT was employed to monitor dedifferentiated disease. This modality enables comprehensive assessment of tumor extension to adjacent structures and detection of metastatic spread, particularly to lymph nodes, with a reported sensitivity of 80-96% for detecting cervical metastases [10,11].

After establishing refractoriness to radioiodine therapy, the patient was treated with sorafenib, dabrafenib, and trametinib. These agents are TKIs that play a key role in modulating cellular growth signals, including tumor cell proliferation, angiogenesis, and metastatic potential [12]. Such targeted therapies aim to stabilize disease or delay progression. For example, sorafenib has been shown to improve progression-free survival in patients with radioiodine-refractory disease [13,14].

## Conclusions

As seen in this patient, FDG-PET/CT enables precise evaluation of metabolic activity, detection of disease progression, and guidance for targeted therapy decisions. It plays a crucial role in the follow-up and management of patients with clinically challenging conditions such as radioiodine-refractory papillary thyroid carcinoma. Continuous technological advances in nuclear medicine imaging contribute to improved diagnostic accuracy and patient outcomes in oncology.
